# Identification and characterization of male reproduction-related genes in pig (*Sus scrofa*) using transcriptome analysis

**DOI:** 10.1186/s12864-020-06790-w

**Published:** 2020-06-01

**Authors:** Wenjing Yang, Feiyang Zhao, Mingyue Chen, Ye Li, Xianyong Lan, Ruolin Yang, Chuanying Pan

**Affiliations:** 1 Key Laboratory of Animal Genetics, Breeding and Reproduction of Shaanxi Province, College of Animal Science and Technology, Northwest A&F University, Yangling, Shaanxi 712100 PR China; 2 College of Life Sciences, Northwest A&F University, Yangling, Shaanxi 712100 PR China; 3 Present Address: Northwest A&F University, No.22 Xinong Road, Yangling, Shaanxi 712100 PR China

**Keywords:** Pig, Transcriptome, Testis-specific genes (TSGs), Male reproduction, Species comparison, Regulatory mechanism

## Abstract

**Background:**

The systematic interrogation of reproduction-related genes was key to gain a comprehensive understanding of the molecular mechanisms underlying male reproductive traits in mammals. Here, based on the data collected from the NCBI SRA database, this study first revealed the genes involved in porcine male reproduction as well their uncharacterized transcriptional characteristics.

**Results:**

Results showed that the transcription of porcine genome was more widespread in testis than in other organs (the same for other mammals) and that testis had more tissue-specific genes (1210) than other organs. GO and GSEA analyses suggested that the identified test is-specific genes (TSGs) were associated with male reproduction. Subsequently, the transcriptional characteristics of porcine TSGs, which were conserved across different mammals, were uncovered. Data showed that 195 porcine TSGs shared similar expression patterns with other mammals (cattle, sheep, human and mouse), and had relatively higher transcription abundances and tissue specificity than low-conserved TSGs. Additionally, further analysis of the results suggested that alternative splicing, transcription factors binding, and the presence of other functionally similar genes were all involved in the regulation of porcine TSGs transcription.

**Conclusions:**

Overall, this analysis revealed an extensive gene set involved in the regulation of porcine male reproduction and their dynamic transcription patterns. Data reported here provide valuable insights for a further improvement of the economic benefits of pigs as well as future treatments for male infertility.

## Background

Pigs (*Sus scrofa*) were amongst the earliest animals to be domesticated and were domesticated from the wild boars approximately 9000 years ago [1]. In comparison with other large livestock, pigs reproduce rapidly, generate large litter sizes, and are easy to feed; these characteristics mean that pigs are of a high economic value in the global agricultural system [1]. Pigs are also an excellent biomedical model for understanding various human diseases (including obesity, reproductive health, diabetes, cancer, as well as cardiovascular and infectious diseases), as pigs and humans are very similar in many aspects of their anatomy, biochemistry, physiology and pathology [2, 3]. Studies have shown that more than half of the cases of childlessness globally were due to male infertility issues, including semen disorders, cryptorchidism, testicular failure, obstruction, varicocele and so on [4–6]. Male infertility affected > 20 million men worldwide and has developed into a major global health problem [5, 6]. Studies have also shown that boar and human spermatozoa had similar courses during fertilization and early embryonic development [7, 8]. These observations mean that research on the pig male reproduction direction is not only the need of the economy, but also can provide insights into human male sterility. It is one of the current research hotspots.

Male reproduction is a complex process that involves cell fate decisions and specialized cell divisions, which requires the precise coordination of gene expression in response to both intrinsic and extrinsic signals [9, 10]. A good deal of recent studies have indicated that the inactivation or abnormal expression of male reproduction-related genes could cause spermatogenesis dysfunction and a decrease in fertility. Studies have also shown that numerous genes related to male reproduction were specifically expressed in the testis of mice or humans, such as *SUN5*, *CFAP65*, *DAZL*, and so on [11–13]. Knockout of the *SUN5* (sad1 and unc84 domain containing 5) gene caused acephalic spermatozoa syndrome and resulted in male sterility in mice [11]. A new homozygous mutation in human *CFAP65* (cilia and flagella associated protein 65) gene has been shown to cause male infertility as it generated multiple morphological abnormalities in sperm flagella [12]. The RNA-binding protein *DAZL* (deleted in azoospermia like) acted as an essential regulator of germ cell survival in mice [13].

As a result of the development of new technologies, especially high-throughput RNA sequencing (RNA-seq), a deeper understanding of mammalian male reproductive regulation genes has been initiated. Developments of high-throughput RNA-seq technology have enabled the accurate and sensitive assessment of transcripts and isoform expression levels [14]. This also means that the transcriptome complexity of more-and-more species has been elucidated and opportunities have been afforded for unprecedented large-scale comparisons across taxa, organs, and developmental stages. However, at the same time, current studies exploring mammalian male reproduction using high-throughput RNA-seq techniques are focused on common model animals such as mice [15]. Large livestock animals such as the pig have received much less research attention to date and have mainly been utilized in order to explore molecular mechanisms associated with pig growth traits such as fat deposition and muscle development [16, 17]. The genes associated with porcine male reproduction and their transcriptional characteristics thus remain unclear, and need to be systematically explored and evaluated.

This study was the first of its kind to explicitly investigate the genes related to porcine male reproduction as well as their transcriptional characteristics. Specifically, this study used five mammalians (pig, cattle, sheep, human and mouse) RNA-seq data to identify testis-specific genes (TSGs) and explore the regulatory mechanisms of TSGs expression. The aim of this research was to address the following questions: 1) What is the extent of genome transcription in different organs for these five mammals? Is the transcription of genes in testis different from that in other porcine tissues? Are porcine TSGs related to male reproduction (i.e., spermatogenesis, germ cell development, spermatid differentiation, and others)? 2) If so, are there some TSGs that are unique for the pig or conserved across species during evolution? What are the expression characteristics of these gene sets and what about the difference between them? 3) What are the factors that regulate and influence the expression of TSGs? What role do alternative splicing, transcription factor binding and gene interactions play in regulating the transcriptional abundance of porcine TSGs? The results of this study augment our understanding of the male reproductive regulation mechanisms in the pig from the perspective of TSG transcription and provide a scientific basis for improving pig reproductive performance and treating male sterility.

## Results

### Widespread protein-coding gene transcription in the mammalian testis

**Fig. 1:**
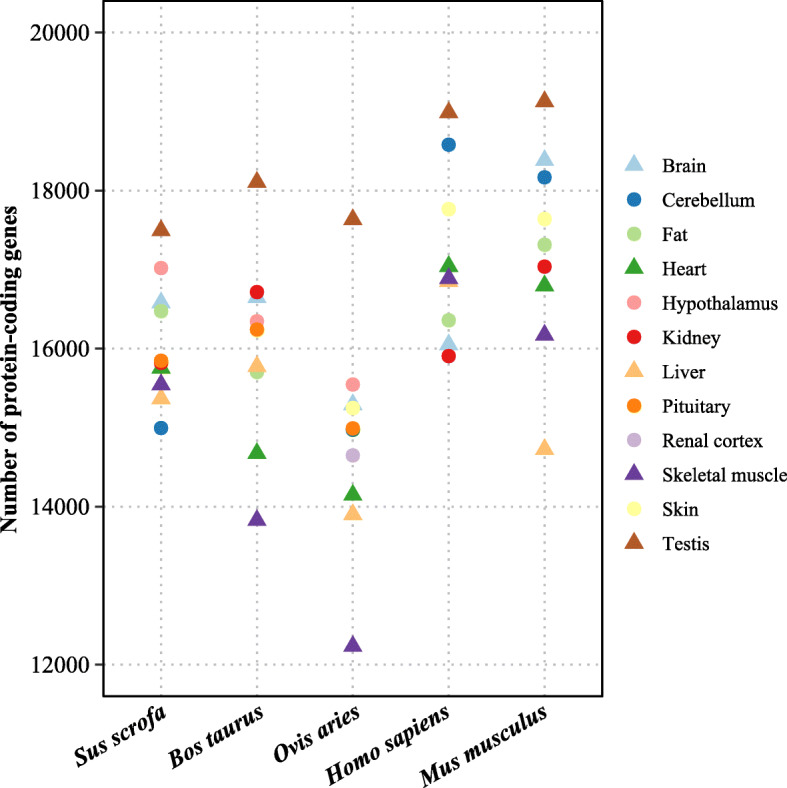
Transcriptome complexity of the mammalian testis. Number of transcribed protein-coding genes in 12 organs from five mammals: pig, cattle, sheep, human and mouse, based on RNA-seq clean reads per sample. Triangles represent common tissues while circles represent non-common organs

To assess the extent of gene transcription in different organs, a RNA-seq data set was used here. This dataset involved 12 organs (testis, brain, cerebellum, hypothalamus, pituitary, heart, liver, kidney, fat, renal cortex, skeletal muscle and skin) of five mammals: pig, cattle, sheep, human and mouse (Table S1). Among them, the transcriptome data of testis, brain, heart, liver and skeletal muscle were available for all the five mammals. The RNA-seq data were mapped onto the reference genome of the corresponding species and resulted in more than 80% average mapping ratio in these species and > 10 million mapped reads of 76 bp per sample (Table S2-S6 and Fig. S1). Analyses of these mammalian data confirmed that protein-coding genes were more frequently transcribed in testis than in other tissues in all the species analyzed (*P* < 8.88× 10^− 8^, chi-square test) (Fig. 1), yielding a pattern consistent with previous estimates for humans, rhesus macaque, mouse, opossum and chicken [18, 19]. Together, testis had high transcriptome complexity.

### Gene expression patterns revealed pig male reproduction-related genes

**Fig. 2:**
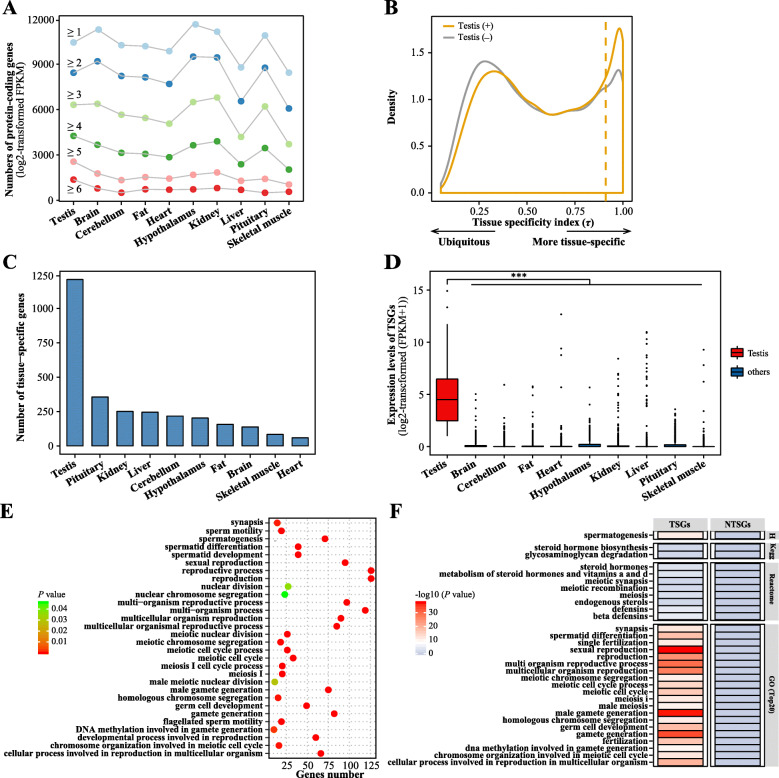
Screening TSGs and revealing genes related to male reproduction in the pig. **a** Distribution of the number of protein-coding genes in various pig tissues with different expression levels (log2 transformed FPKM). Note: colour code is palette = “paired”. **b** Distribution of the tissue specificity index (*τ*) of protein-coding genes across ten or nine (except testis) tissues is showed. The dotted line represents the value of the top 20% of the tissue specificity index scores. **c** Number of tissue-specific genes in the various tissues. **d** Boxplots show the expression level of TSGs in testis and nine other tissues. The significance level is determined using one-sided Wilcoxon rank-sum test (*P* < 2.00 × 10^− 16^). * *P* < 0.05; ** *P* < 0.01; *** *P* < 0.001. **e** GO analysis for TSGs. **f** Heat map showing the enriched gene sets for porcine TSGs based on hypergeometric distribution test. NTSGs, non-testis-specific genes; H, hallmark gene sets; KEGG, Kyoto Encyclopedia of Genes and Genomes gene sets; GO, Gene Ontology gene sets

The pig was used as the model system in this study in order to explore the high transcription complexity seen in testis. Results showed that protein-coding gene expression levels vary across tissues and testis had a distinct distribution (Fig. S2). Among them, as expression level increased, the proportion of genes with high expression levels (log_2_ FPKM ≥4) in testis gradually increased compared to other tissues (Fig. 2A).

The results of previous study demonstrated that many genes related to male reproduction were specifically expressed in the testis (Table S7) [11–13]. Therefore, in order to further elucidate genes that were associated with male reproduction in pigs, TSGs were investigated using the distribution of the tissue specificity index *τ*. Interestingly, data showed that testis contributed considerably to tissue specificity, and the number of tissue-specific genes in the testis was far higher than in others (such as brain, liver, heart and so on) (Fig. 2B, C).

A total of 1210 TSGs were obtained from pig when the *τ* score was greater than the top 20% value of *τ* (*τ* value ≥ 0.91) (Fig. 2B-D and Table S8). TSG expression levels in testis were significantly higher than those in other tissues (*P* < 2.00 × 10^− 16^) (Fig. 2D). GO functional analysis revealed that these TSGs were significantly enriched for functions associated with male reproduction, including sperm motility, spermatogenesis, sperm development, reproduction and so on (Fig. 2E). GSEA also showed that these TSGs were involved in gene sets and signal pathways related to male reproduction (Fig. 2F).

### Characterizing unique or conserved during evolution TSGs in the pig

Several studies have highlighted that there were differences in gene expression levels between species, yet some tissues (such as testis, brain, heart, etc.) usually have conserved gene expression patterns [20–22]. We therefore proposed a hypothesis that TSGs of the pig might also be testis-specific in other phylogenetically closely related species (genetic relationship was revealed using TimeTree website [23]), such as cattle, sheep, human and mouse. To verify this assumption, 13,253 orthologous gene families and 10,740 1: 1 orthologous genes were first identified in these five mammals (Fig. S3).

**Fig. 3:**
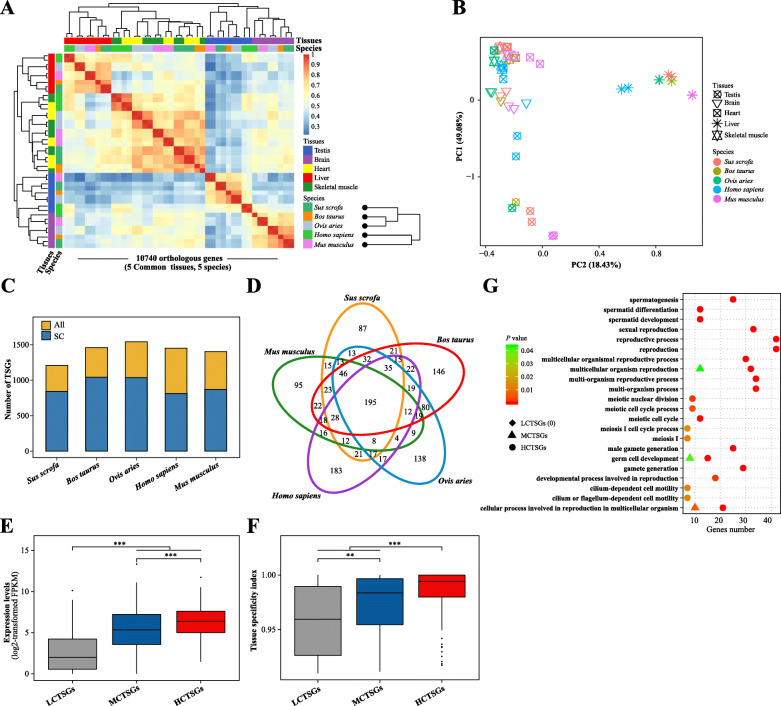
Comparison of unique or conserved TSGs in the pig using cross-species analysis. **a** Clustering of samples based on expression values, FPKM of singleton orthologous genes present in all five species (*n* = 10,740) are calculated. Single linkage hierarchical clustering is used. (Bottom right) Phylogenomic relationships of the five mammals. **b** Factorial map of the principal component analysis of expression levels for 1:1 orthologous gene. The proportion of variance explained by the principal components is indicated in parentheses. **c** Bar charts represent the number of all TSGs (All) and single-copy TSGs (SC) in each mammal. **d** Number of unique TSGs and conserved TSGs in the pig. The 10,740 1:1 orthologous gene identified are used as a reference. **e-f** Comparison of expression levels in testis (**e**) and tissue specificity index scores (**f**) between LCTSGs (*n* = 87), MCTSGs (*n* = 113) and HCTSGs (*n* = 195), respectively. The statistical test in the panel is based on the one-sided Wilcoxon rank-sum test. * *P* < 0.05; ** *P* < 0.01; *** *P* < 0.001. **g** Functional annotation of the three gene sets (LCTSGs, MCTSGs and HCTSGs) in the pig

Then, based on the FPKM values of the 10,740 orthologous genes, pearson correlation coefficients for common tissues from five mammals were calculated, and cluster analysis and principal component analysis (PCA) were performed. The results showed that the gene expression pattern between homologous tissues of different species was more similar than that between different tissues of the same species and that the replicates within each sample exhibited high reproducibility (Fig. 3A, B).

A similar analysis was then also performed to calculate TSGs using organ RNA-seq data from the four additional mammals, and found that the number of TSGs in cattle, sheep, human and mouse were 1459, 1541, 1403 and 1452, respectively (Fig. 3C and Fig. S4). Next, on the basis of a gene family size, genes were classified as single-copy genes (SC) and multi-copy genes (MC, gene family size ≥2). The TSGs of each species were mostly single-copy genes (Fig. 3C). Meanwhile, based on the correspondence of 10,740 1:1 orthologous genes between the five mammals, Fig. 3D showed 195 TSGs with high expression conservation (HCTSGs, shared by all five species), 113 TSGs with moderate expression conservation (MCTSGs, shared by pig, cattle and sheep) and 87 TSGs with low expression conservation (LCTSGs, unique to pig) in pig (Fig. 3D and Table S8).

Also, the expression levels and tissue specificity index scores between LCTSGs, MCTSGs and HCTSGs in the pig were compared, respectively. These comparisons showed that HCTSGs exhibited significantly greater expression levels and tissue-specific index scores than either MCTSGs or LCTSGs and that there were differences in the functions of these three gene sets (Fig. 3E-G). Indeed, the more conservative the gene expression level, the easier it was for a gene to become enriched for male reproduction-related functions (Fig. 3G).

### Evolutionary rates of porcine TSGs were relatively higher

Due to differences in selective pressures, the evolutionary rates of gene expression vary between organs and lineages, and these variations were thought to be a basis for the development of phenotypic differences of many organs in mammals [24]. Thus, we assessed how the TSG evolutionary rate in the pig had changed.

**Fig. 4:**
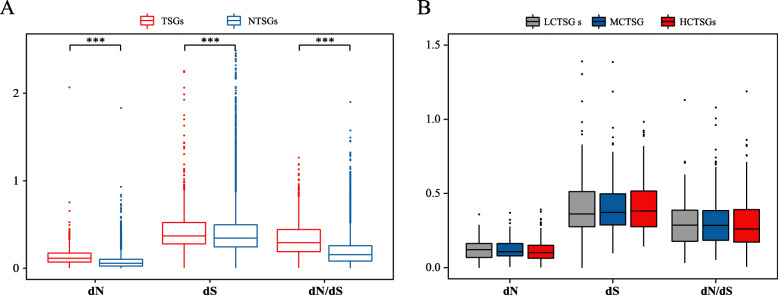
Evolutionary rates of TSGs in the pig. **a** Distribution patterns of TSGs and NTSGs in pig based on the value of dS, dN and dN/dS (evolutionary rate), respectively. **b** dS, dN and dN/dS values between the three gene sets of LCTSGs, MCTSGs and HCTSGs are compared, respectively. All the statistical tests in the panel are based on the one-sided Wilcoxon rank-sum test. * *P* < 0.05; ** *P* < 0.01; *** *P* < 0.001

Compared with NTSGs, porcine TSGs were found to have significantly higher dN, dS and gene evolutionary rate (dN/dS) (Fig. 4A). At the same time, however, although there were no significant differences in the rate of evolution between LCTSGs, MCTSGs and HCTSGs sets, highly expressed conserved TSGs nevertheless had a relatively low evolutionary rate and were more conserved (Fig. 4B).

### Porcine TSGs alternative splicing patterns

**Fig. 5:**
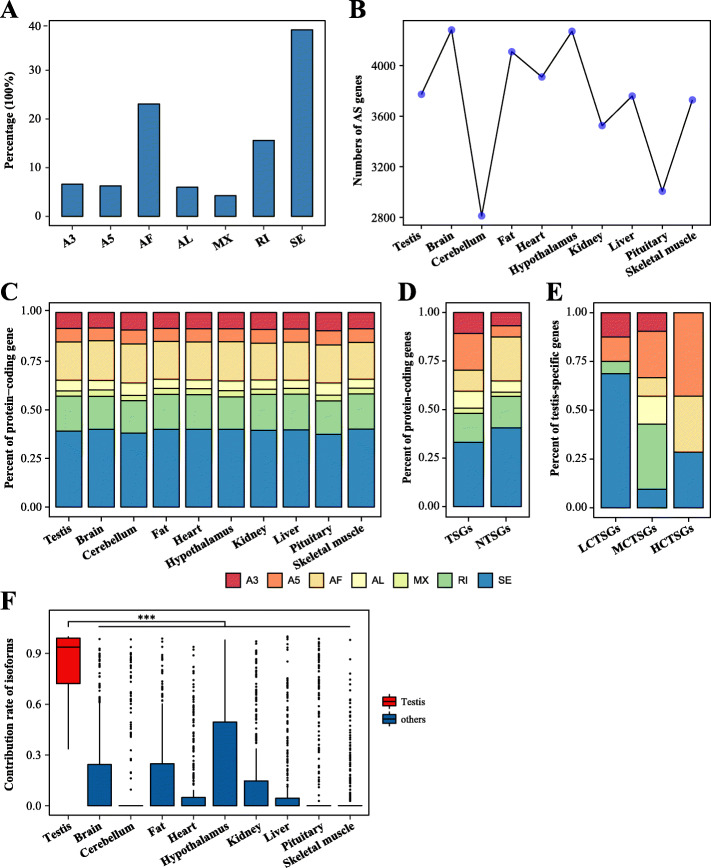
Characterization of dynamic patterns of alternative splicing and its regulation in TSGs of the pig. **a** Proportion of protein-coding genes affected by various AS event types. A3, alternative 3′ splice sites; A5, alternative 5′ splice sites; AF, alternative first exons; AL, alternative last exons; MX, mutually exclusive exons; RI, retained intron; SE, exon skipping. **b** Number of protein-coding genes affected by AS in each tissue type. **c** Stacked bar plot indicates the distribution ratio of protein-coding genes with different splicing events in each tissue type. **d** The proportion of AS events changes between TSGs and NTSGs in the pig. **e** Differences in the distribution of genes with various splicing events between LCTSGs, MCTSGs and HCTSGs in the pig. **f** For testis and other nine tissues, the contribution rate (FPKM_isoform_ / (FPKM_TSG_ + 1)) of the most highly expressed isoforms to TSGs with multiple isoforms (≥ 2). The statistical test in the plot is based on the one-sided Wilcoxon rank-sum test. * *P* < 0.05; ** *P* < 0.01; *** *P* < 0.001

The achievement of different functions for genes in different tissues and cells required the process: alternative splicing (AS), which would lead to changes in gene expression and thus change phenotype [25]. To clearly illustrate the complex AS patterns of porcine TSGs, 23,059 AS events (including SE, IR, A5, A3, MX, AF and AL) were identified, which correspond to 8027 protein-coding genes. The data presented in Fig. 5A revealed that the major splicing pattern in porcine protein-coding genes was exon skipping (Fig. 5A). Remarkably, more protein-coding genes (3772) had splice variants in testis than in certain organs (cerebellum, kidney, liver, pituitary and skeletal muscle) (Fig. 5B).

This study then determined the distribution of genes affected by seven AS events in each porcine tissue, and found that trends in the distribution of these AS events were basically consistent in all analyzed tissues and the SE remained the major splicing event (Fig. 5C). This study further identified AS changes between porcine TSGs and NTSGs, the most frequent changes were the number of TSGs in which A5, AF, and SE events occurred (Fig. 5D). Moreover, the study explored changes in the splicing pattern of TSGs with diverse degrees of conservation (LCTSGs, MCTSGs and HCTSGs). The distribution of splicing events in these three gene sets was completely different, and these TSGs were affected by different splicing types (Fig. 5E).

It was clear that a range of different gene isoforms was produced by AS in testis, and we speculated whether the highly expression genes were the result of the high expression of certain transcript isoforms. Hence, the isoform contribution rates with highest expression in testis to the expression of TSGs were calculated. Among TSGs with multiple transcripts, the median number of contribution ratio per gene was 0.937, supporting our conjecture (Fig. 5F). At the same time, Fig. 5F showed that this phenomenon was significantly reduced in other organs (*P* < 2.00 × 10^− 16^) (Fig. 5F).

### Transcriptional control in porcine TSGs

**Fig. 6:**
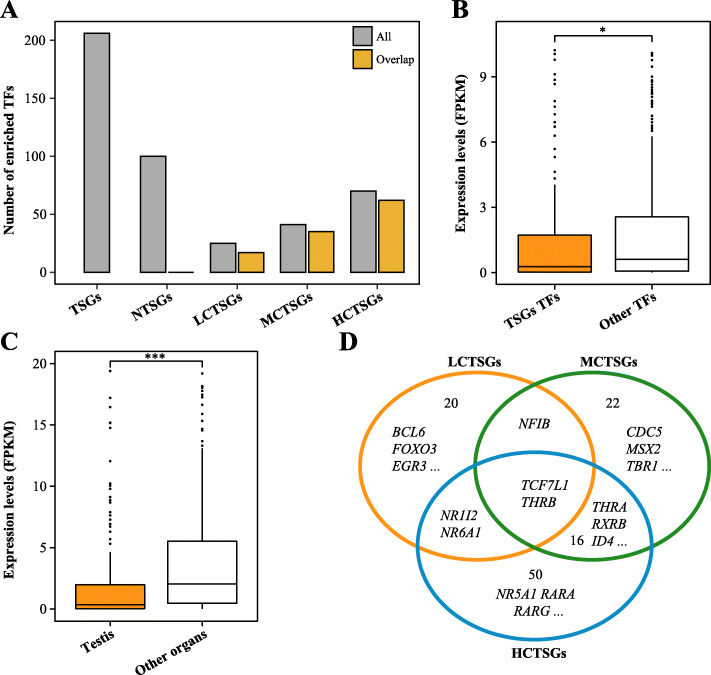
Transcriptional regulation in porcine TSGs. **a** Bar graphs illustrate the number of TFs associated with TSGs, NTSGs, LCTSGs, MCTSGs and HCTSGs, respectively. All, the number of TFs associated with each gene set of the pig; Overlap, the number of overlap of TFs identified from TSGs and those identified from other gene sets. **b** Differences in the expression levels of enriched and unenriched TFs of TSGs in testis. **c** Expression levels of enriched TFs of TSGs in testis and other tissues (except for testis, the average FPKM of other remaining nine tissues). **d** Venn diagram showing shared or differential TFs between LCTSGs, MCTSGs and HCTSGs. All statistical tests in the panel are based on the one-sided Wilcoxon rank-sum test. * *P* < 0.05; ** *P* < 0.01; *** *P* < 0.001

Transcription factors (TFs) are proteins that bind to specific DNA sequences, influence the expression of neighboring or distal genes, and are a central determinant of gene expression [26]. One of the aims of this study was to evaluate which TFs regulate porcine TSGs. The results presented here showed that 206 TFs were significantly associated with TSGs and not to NTSGs, and these TSGs were preferentially regulated by TFs such as *AR*, *THRB*, *NR5A1*, *SOX9* (Fig. 6A and Table S9). Furthermore, TSGs-related TFs were expressed at lower abundance than that of its unrelated TFs in testis (*P* = 0.014) (Fig. 6B). Data also showed that the abundance of TSGs-related TFs in testis remained significantly lower than its average abundance in the other nine tissues (*P* = 1.7 × 10^− 9^) (Fig. 6C).

This study tested whether there were essential TFs that regulate TSGs expression, as determined by the differences in TFs enrichment between LCTSGs, MCTSGs and HCTSGs. Interestingly, although the number of TFs associated with these gene sets was disparate, they overlapped significantly with those identified in whole TSGs at ratios of 68%, 85.4%, and 88.6%, respectively (Fig. 6A and Table S9). Beyond that, the analysis predicted that *TCF7L1* (transcription factor 7 like 1) and *THRB* (thyroid hormone receptor beta) might play a crucial regulator role for TSGs, whereas many other TFs could also potentially regulate the expression abundance of TSGs in the pig (Fig. 6D).

### Establishing gene regulation network of porcine TSGs

**Fig. 7:**
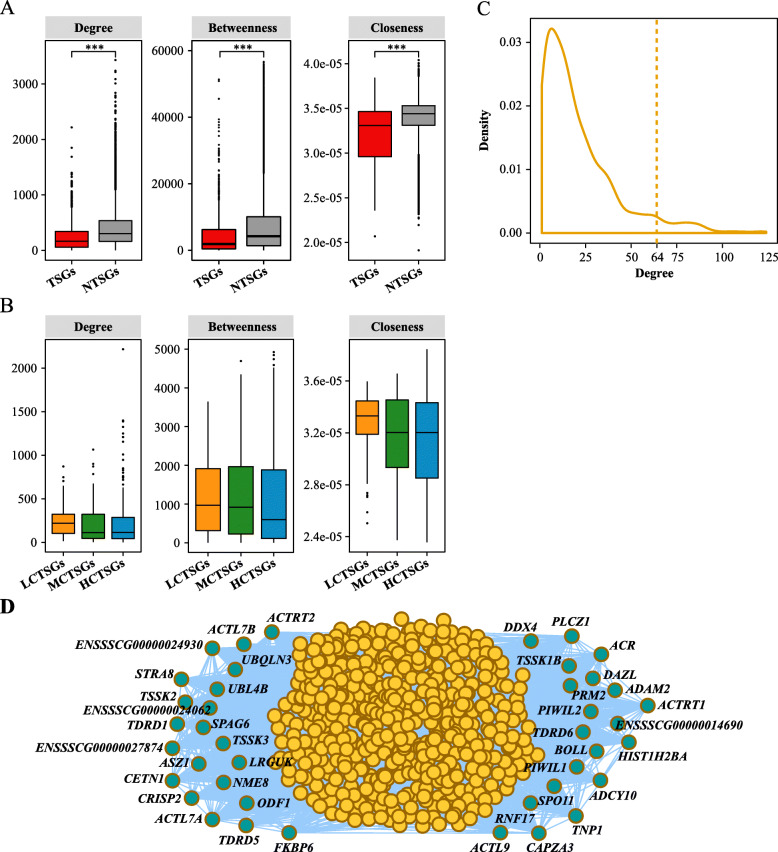
Gene regulation network analysis of TSGs in the pig. **a** Degree centrality, betweenness centrality and closeness centrality between TSGs and NTSGs in pig. **b** Degree centrality, betweenness centrality and closeness centrality between LCTSGs, MCTSGs and HCTSGs in the pig. *P* values are calculated using one-sided Wilcoxon rank-sum test. * *P* < 0.05; ** *P* < 0.01; *** *P* < 0.001. **c** Density plot showing the distribution of degree centrality between 1210 porcine TSGs. The dotted line represents the value of top 5% of degree centrality. **d** Gene regulation network of TSGs. The edges (blue lines) indicate interactions between TSGs, while circles indicate each TSG. Blue circles indicate the hub TSGs. Only hub TSGs and their interaction genes are shown in the plot

Simple linear connections between organismal genotypes and phenotypes do not exist. It was clear that the relationships between most genotypes and phenotypes were the result of much deeper underlying complexity [27, 28]. The regulation network of TSGs in the pig was therefore explored in this analysis. Data showed that the degree centrality, betweenness centrality and closeness centrality were significantly lower in TSGs when compared to NTSGs (Fig. 7A). It was also noteworthy that these three centralities were not significantly different between LCTSGs, MCTSGs and HCTSGs (Fig. 7B).

This study also evaluated TSGs that play a central regulatory role in the regulation of male reproduction of pig, employing the regulation network between TSGs (including 767 nodes and 8071 edges). Thus, on the basis of the distribution of degree centrality, 41 candidate hub TSGs were screened within the TSGs regulatory network and a top 5% value of degree centrality was used as a threshold (Fig. 7C, D). Some examples of genes that passed this screen included *DAZL*, *CAPZA3* (capping actin protein of muscle Z-line subunit alpha 3), *STRA8* (stimulated by retinoic acid 8), *DDX4* (DEAD-box helicase 4), and so on (Fig. 7D).

### Verifying TSG abundances in the pig by qRT-PCR

**Fig. 8:**
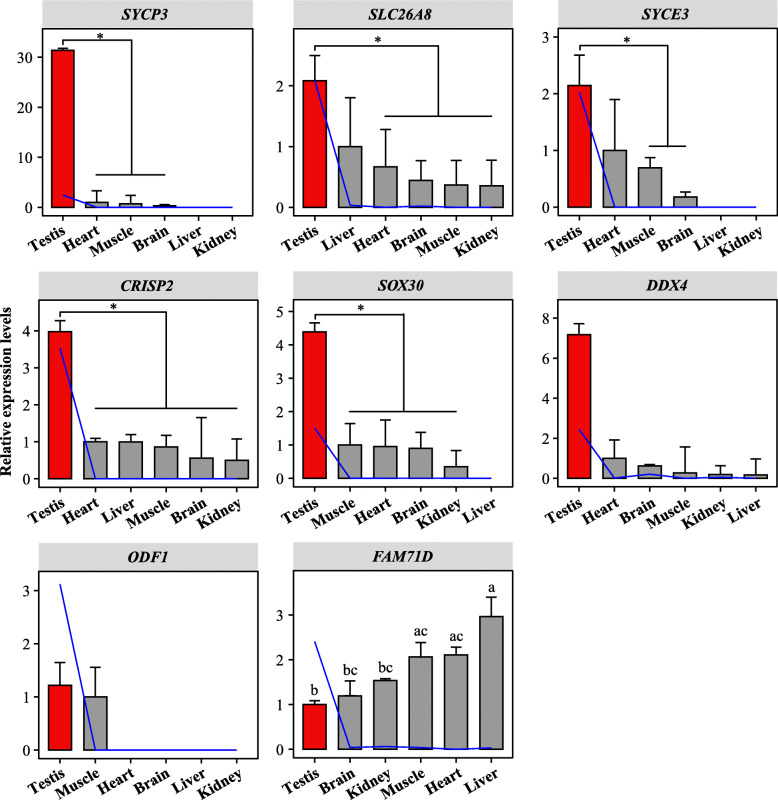
Validation of the expression levels of porcine TSGs using qRT-PCR. Histograms show mRNA expression levels of eight TSGs randomly selected in different tissues (including testis, brain, heart, liver, kidney and muscle) of LY pig (*n* = 3). The blue line represents the results of the RNA-seq analysis (expression levels: log_10_(FPKM+ 1) transformed). Ct values above 35.0 are considered not biologically significant in this study, and tissue values are not shown in these cases. Data represent Mean ± SEM. * *P* < 0.05; Values with different letters (**a**-**c**) differ significantly at *P* < 0.05

To verify the results of the RNA-seq data analysis, qRT-PCR was performed on eight randomly selected TSGs of LY (Landrace×Yorkshire) pig. The results presented in Fig. 8 showed that qRT-PCR analysis was performed using total mRNA from testis, brain, heart, liver, kidney and muscle. Seven genes with testis-specific expression were observed (including *SYCP3* (synaptonemal complex protein 3), *SLC26A8* (solute carrier family 26 member 8), *SYCE3* (synaptonemal complex central element protein 3), *CRISP2* (cysteine rich secretory protein 2), *SOX30* (SRY-box transcription factor 30), *DDX4* and *ODF1* (outer dense fiber of sperm tails 1)) in this analysis, except for *FAM71D* (family with sequence similarity 71 member D), consistent with the findings of the RNA-seq data (Fig. 8).

## Discussion

Individual protein-coding gene studies performed since 2000 have reported on the presence of unique transcription patterns in testis and subsequent effects on reproduction [29]. In one example, *Sox30* was highly expressed in mice spermatocytes and spermatids and the associated knockout line was sterile [30]. Despite the clear biological importance of male reproduction, our current knowledge regarding the underlying molecular mechanisms of protein-coding gene regulation in mammalian reproduction remains largely incomplete. Amongst the numerous available methods for studying gene expression and function, RNA-seq has become preferred [14]. In this study, transcription patterns in porcine testis as well as four other phylogenetically related mammals were evaluated using RNA-seq data. The results revealed that substantially more widespread transcription of protein-coding genes in testis than in other tissues for all species. And, combined with earlier Soumillon reports, these results suggested that the complex transcriptional activity in testis was common across mammals [19].

Further transcriptome analyses of all major organs in the pig revealed that testis had a high proportion of genes with high expression levels (log_2_ FPKM ≥4) compared with other tissues. This potentially reflected specific functional requirement. Additionally, numerous genes related to male reproduction were specifically expressed in testis, such as *SUN5*, *CFAP65*, *DAZL* [11–13]. Therefore, in order to explore genes associated with male reproduction in pigs, 1210 TSGs were screened in this study using the tissue specificity index. GO and GSEA found that these genes were significantly associated with germ cell development. Indeed, the androgens-related genes *CYP11A1* and *HSD17B3* were specifically expressed in pig testis, which affected testosterone secretion and thereby regulated spermatogenesis [31, 32]. In other words, these results confirmed the accuracy of analysis in this study. Interestingly, these TSGs were preferentially located on the sex chromosome, especially the X chromosome (X-chromosome: *P* = 8.73 × 10^− 12^; Y-chromosome: *P* = 1.11 × 10^− 9^, one-sided Wilcoxon rank-sum test) (Fig. S5). This might be related to the function of the testis, which produces sperm, transmits genetic information and maintains species continuance [33]. TSGs in the pig evolved more rapidly than NTSGs because of the differences in selective pressure [34]. As a kind of gonadal organ, testis was influenced by positive selection of evolutionary forces associated with sperm competition and other sex-related factors [33, 34]. The data also indicated that testis contributed considerably to tissue specificity of gene expression and the number of tissue-specific genes in testis was far more than that of other tissues. These apparent differences might be due to the gene expression changes in testis, which was the most rapidly divergent throughout evolution [21, 24].

Notably, utilizing 1:1 orthologous genes from the five mammals tested, this study further systematically investigated and uncovered the transcriptional characteristics of porcine TSGs that expression conserved (such as *SPESP*, *SLC26A8* and so on). *SPESP1*, sperm equatorial segment protein 1 was involved in normal spermatogenesis and influenced sperm formation in mouse [35]. It was also clear that the missense mutation in *SLC26A8* was associated with human asthenozoospermia [36]. The breadth of expression across tissues was referred to as pleiotropy and, in particular, expression pleiotropy was correlated with phenotypic impact in mammals [37]. In line with this, the results of this study showed that HCTSGs exhibited relatively higher expression abundance and tissue specificity and were more likely associated with male reproduction. Furthermore, compared to LCTSGs and MCTSGs, a lower evolutionary rate in HCTSGs suggested that their expression levels might be subject to larger evolutionary constraint. These results suggested that pleiotropy exerted a potential influence on gene evolution [38].

Several mechanisms could be invoked to explain specific transcription in porcine TSGs. Initially, analyses uncovered extensive dynamic processes of alternative splicing in TSGs, underscoring the importance of post-transcriptional regulation. Changes in A5, AF and SE events were the predominant features of splicing regulation of porcine TSGs. The study also detected 1569 testis-specific transcripts (Table S10). Interestingly, high expression of TSGs was caused by high expression of certain isoforms, and its median contribution rate in the testis was 93.7%. This contribution rate was significantly reduced in other tissues and isoforms with high expression abundance in different tissues were altered, further demonstrating that AS was involved in the regulation of gene expression of TSGs. In addition, earlier work has shown that TFs were vital in gene regulatory networks [26, 39]. Transcriptome analysis was used here to define key TFs in porcine TSGs regulation, including *TCF7L1* and *THRB*. Results also reflected that most TFs had a potentially negative regulatory effect on TSG expression. One component of the Wnt signaling pathway, *TCF7L1*, controlled pluripotent stem cell (such as primordial germ cell) self-renewal and lineage differentiation [40]. Similarly, *THRB* was a receptor for the thyroid hormone *TH*, and *TH* involved in controlling the proliferation and differentiation of Sertoli cells and Leydig cells during testicular development in mammals [41]. As genes often function together, synergistic expression changes of distinct genes might be phenotypically relevant [24, 27]. The outcomes of this study revealed that TSG functional similarities resulted in preferences for interactions between these rather than other protein-coding genes. 41 hub TSGs (such as *CAPZA3*, *DAZL*, *STRA8* and so on) in the pig played crucial roles, which directly interacted with the majority of other testis-specific candidate genes and might be involved in regulatory cascades related to male reproduction. Amongst these, *CAPZA3* was located in sperm cells and its mutations caused the sperm mutant phenotype of repro32 infertile male mice [42]. *Stra8* was required for meiotic initiation in mice and also promoted spermatogonial differentiation [43]. These observations all suggested that TSG transcription was regulated by a range of factors, part of the regulation process of testis complexity. However, the extent to which each factor contributed remained unclear and would require further investigation.

Taken together, this study has yielded precise, previously uncharacterized, and specific molecular features of TSGs in the pig. This will not only enrich our understanding of male reproductive regulation mechanisms in pigs, but also provide a scientific basis for improving the reproductive performance of the pig and treating male sterility. And the next challenge we face will be to determine how these genes associated with male reproduction function. This can be combined with other massive parallel sequencing technologies (such as single-cell transcriptomics and epigenomics datasets) for further dissection of the complex molecular regulation networks, including dynamic epigenetic features and post-transcriptional regulation mechanisms.

## Conclusions

In conclusion, complex transcriptional activity in testis was common in mammals. Transcriptomic analysis, undertaken here for the first time, identified a total of 1210 porcine TSGs (including 87 LCTSGs, 113 MCTSGs and 195 HCTSGs), and these genes are related to male reproduction. Moreover, alternative splicing, transcription factors and other TSGs all regulate TSGs expression. Thus, the findings reported here contribute to a better understanding of the molecular regulation mechanisms underlying male fertility in the pig and provide a scientific basis for enhancing the economic benefits of this species as well as treating male sterility.

## Methods

### Data collection

The study compiled a total of 88 transcriptome datasets from various organs (testis, brain, cerebellum, hypothalamus, pituitary, heart, liver, kidney, fat, renal cortex, skeletal muscle and skin) in pig (*Sus scrofa*, *n* = 10), cattle (*Bos taurus*, *n* = 4), sheep (*Ovis aries*, *n* = 2), human (*Homo sapiens*, n = 10) and mouse (*Mus musculus*, *n* = 7). Considering sample batch, the number of reads, the quality of the sequencing, and the consistency with the data from other tissue datasets, each tissue obtained 2–3 datasets for subsequent analysis. It is a special case that the tissues of some species retain only one sample. Between 15 and 20 datasets were downloaded for each specie, and these data were downloaded from the NCBI SRA database (https://www.ncbi.nlm.nih.gov/sra) (Table S1). The transcriptome data for testis, brain, liver, heart and skeletal muscle were available for all the five species.

### RNA-seq data processing

First, the quality of raw reads was evaluated using FastQC software (version 0.11.7) [44]. Low quality reads and adaptor sequences were removed using Trimmomatic (version 0.38) [45]. Furthermore, we cut the nucleotides at both ends of a read until the remaining part (clean reads) was 76 bp in length. Second, the clean reads were then mapped to the corresponding reference genome using HISAT2 (version 2.1.0) program [46]. Only the best hits were retained for the quantification of gene expression level (−k 1). At last, the transcript abundance of known protein-coding genes was quantified as fragments per kilobase of transcript per million mapped reads (FPKM) using Stringtie (version 1.3.4d) [46]. Gene-level FPKM was calculated as the sum across all its isoforms. The reference genome sequences (assembly) and GTF annotations for these five mammals were downloaded from the Ensembl genome browser 92.

### Identification of tissue-specific genes

To identify testis-specific genes, we calculated the tissue specificity of each gene. For this purpose, we first discarded the non-expressed genes with expression level equal to zero in all tested tissues. Then the tissue specificity index τ for each gene was measured based on a previously described strategy [47, 48]: $=\frac{\sum_{i=1}^N\left(1-\frac{\log_2\left({x}_i+1\right)}{\log_2\left({x}_{max}+1\right)}\right)}{N-1}$, where *N* denotes the number of organs to be sampled in a species, and *x*_*max*_ represents the maximum expression of gene *i* across the *N* organs. The values of *τ* lie between 0 and 1. A score close to 1 indicates that the gene is highly tissue-specific, while a score close to 0 indicates that this gene is ubiquitous in all tested tissues. In the case of tissue samples with multiple biological replicates, FPKM values for genes were averaged. Based on the distribution of *τ* scores, genes with the top 20% of *τ* were selected as the candidate tissue-specific genes. After that, the genes specifically expressed in each tissue type were further refined with the following criteria: FPKM ≥1 in a given tissue, and the expression level of a gene in a given tissue ranks top 3 in the inspected tissues [49].

### Gene ontology (GO) and gene set enrichment analysis (GSEA)

To infer the putative functions of porcine TSGs, we conducted GO (http://geneontology.org/) enrichment analysis [50]. Significantly enriched GO terms were defined only if their *P* value (corrected *P* value) < 0.05.

For GSEA, the various gene sets were retrieved from the GSEA database (http://software.broadinstitute.org/gsea/index.jsp) [51]. And the GSEA data for the pig were built using pig-human 1:1 orthologous gene (detailed information was shown below). Hypergeometric distribution test was then used to calculate significantly enriched gene sets, again using *P* value < 0.05 as the threshold.

### Identification of orthologous genes

Protein annotation files (from the Ensembl genome browser 92) were first filtered to obtain high-quality protein sequence data to identify orthologous genes between multiple species. Initially, the study retained only the longest protein sequence for each protein-coding gene. OrthoMCL (version 2.0.9) was then used to remove protein sequences that were less than ten amino acids in length and had a stop codon ratio greater than 20% [52]. A total of 105,222 protein sequence files for five species following filtering were then used to identify orthologous genes by OrthoMCL. The two key steps were as follows: (1) all-v-all BLAST, which compared each protein to all other proteins in the blast database created by blastp (version 2.7.1) so that two-paired proteins of *E*-value < 1.00 × 10^− 5^ were output; (2) paired proteins were clustered using Markov Cluster algorithm (MCL) to generate final orthologous gene families. The expansion coefficient for MCL clustering was set to 1.5. Among them, 1:1 orthologous genes were defined as conserved, single copy orthologous genes per species.

### Evolution rate of porcine TSGs

The sheep was used as the control. According to the pig-sheep 1:1 orthologous gene pair, values for nonsynonymous (dN) and synonymous (dS) substitution rates of pig were downloaded and compared from the BioMart website (http://asia.ensembl.org/biomart).

### Definition of alternative splicing (AS) events

SUPPA2 (version 2.3), fast and accurate software for identifying all possible AS events [53]. Seven AS events were detected and quantified, including exon skipping (SE), retained intron (RI), alternative 5′ splice sites (A5), alternative 3′ splice sites (A3), mutually exclusive exons (MX), alternative first exons (AF) and alternative last exons (AL). AS events were considered to be present in a tissue if 5 ≤ PSI (Percent spliced in) ≤ 95 [22].

### Porcine genome TFs enrichment test

Binding specificities of TFs were represented by position weight matrices (PWMs). Thus, PWM data for 490 porcine TFs were downloaded from Cis-BP database of Meme Suit website (http://meme-suite.org/db/motifs). To predict potential targets of TFs, the promoter regions (+/− 500 bp around transcription start sites) for all porcine protein-coding genes were scanned by the FIMO (default parameter) (version 4.11.4) [54]. For each transcription factor, an analysis based on the hypergeometric distribution test was used to reveal whether, or not, a gene set was significantly regulated relative to another, using *P* value < 0.05 as the threshold.

### Construction of a gene interaction network

To explore the interaction between genes specifically expressed in pig testis, porcine protein interaction data were downloaded from the STRING (version 11.0) (https://string-db.org/) and BioGRID databases (version 3.5) (https://thebiogrid.org/) [55, 56]. Duplicated and self-ligation interaction data were removed so that 14,049 nodes (genes), 2,752,439 edges (interactions) comprised the background network. The R package: igraph (version 1.2.4) was then used to calculate the degree centrality, betweenness centrality and closeness centrality of the genes [57]. Cytoscape (version 3.7.1) was used to construct a gene interaction network and to screen for interactions between porcine TSGs [58].

### Pig tissue material

In this study, 5 tissues of 3 unrelated seven-day old individuals of male Landrace×Yorkshire (LY) pigs, including testis, brain, heart, liver, kidney and muscle, were randomly sampled from the Besun agricultural industry group Co., Ltd. (Yangling, Shaanxi, China). Animal handling and sampling protocols were in accordance with institutional guidelines for the Faculty Animal Policy and Welfare Committee of Northwest A&F University (FAPWC-NWAFU). The animals were sacrificed by bleeding of carotid artery under sodium pentobarbital anesthesia. Then, fresh tissues were immediately frozen in liquid nitrogen and stored at − 80 °C until used for any other analysis.

### Total RNA isolation and cDNA synthesis

According to the manufacturer’s protocol, Trizol reagent (Takara, Dalian, China) was used to lyse the cells, release nucleic acids, and extract total RNA from the collected tissue samples. cDNA could be synthesized when the OD_260_ /OD_280_ ratio of RNA was in the range of 1.8 to 2.0. The cDNA was synthesized by reverse transcription PCR using Prime Script™ RT Reagent kit (Takara, Dalian, China). Finally, the resultant cDNA was preserved at − 20 °C.

### Porcine TSGs validation using quantitative real-time PCR (qRT-PCR)

In order to quantitatively determine the reliability of data analyzed in this study, eight porcine TSGs were randomly selected to test expression levels. The *β-actin* gene was used as an endogenous control to normalize test gene expression levels and qRT-PCR primers for all genes were designed using the NCBI Primer-BLAST (Table 1). Primers covered different exons to ensure cDNA amplification and expression profiles were performed using the Bio-Rad CFX96 Real-Time PCR system (Hercules, CA, United States).

**Table 1:** The qRT-PCR primer sequences of TSGs in the pig

Gene	Primer sequences (5′-3′)	Sizes (bp)
*SYCP3*-F	CATCGAAGCGAACACCTCAG	150
*SYCP3*-R	GACATCCTCCTCTGAACCACTC	
*SLC26A8* -F	TTGCATCTCTGGTAAGCGCA	121
*SLC26A8* -R	AGGTAGGGTAGGACGTTGCT	
*SYCE3*-F	AATCTCAGTGCAGGCCACG	121
*SYCE3*-R	CTCCATCTCCTCCTTGCAGT	
*CRISP2*-F	CTGCTGTGCTGCTACCATCT	138
*CRISP2*-R	TGGCAGGTGGAGAGACTGAT	
*SOX30*-F	TCGCTCACCTACCTACTCTGT	127
*SOX30*-R	AAGTGTGATGGGACTTGGGC	
*DDX4*-F	TGGGTGGTTTTGGTGTTGGA	153
*DDX4*-R	AGCCGCCTCTCTTGGAAAAA	
*ODF1*-F	GGAAAGCCATCCGAGCCATA	176
*ODF1*-R	CACACACACCTTTCCGTCCT	
*FAM71D*-F	ACGACATCTGCATCCCAGAC	183
*FAM71D*-R	AAGAGCAGATGCCCACAGTC	
*β-actin*-F	CTCCATCATGAAGTGCGACGT	114
*β-actin*-R	GTGATCTCCTTCTGCATCCTGTC	

The 13 μL qRT-PCR reaction contained 5 μL cDNA (1/50 dilution), 0.5 μL of each primer (F/R), 7 μL 2 × SYBR Green (High ROX, BioEasy Master Mix, BIOER). The amplification cycle was as follows: initial denaturation for 1 min at 95 °C for 1 cycle, followed by 39 cycles at 95 °C for 15 s, 60 °C for 15 s and then 72 °C for 30 s. The 2^−ΔΔCt^ method was used to transform Ct values, and the data were compared by one-way ANOVA method using SPSS (Version 19.0).

## Supplementary information

**Additional file 1: Table S1.** SRA samples and project numbers of twelve organs in five mammals. **Table S2.** Mapping statistics of different organs of pig (*Sus scrofa*). **Table S3.** Mapping statistics of different organs of cattle (*Bos taurus*). **Table S4.** Mapping statistics of different organs of sheep (*Ovis aries*). **Table S5.** Mapping statistics of different organs of human (*Homo sapiens*). **Table S6.** Mapping statistics of different organs of mouse (*Mus musculus*). **Table S7.** Examples of identified TSGs presented in Table S8. **Figure S1.** Mapped ratio of mapped to the reference genome in five mammals. **Figure S2.** Distribution difference of protein-coding genes between testis and other organs in pig. (A-I) Quantile-Quantile (q-q) Plots represent the distribution difference of all expressed protein-coding genes (log2-transformed FPKM) between the testis and other nine organs in pig. The blue lines (y = x) mean the same distribution. **Figure S3.** Identification of homologous families and homologous genes in five mammals. (A) Venn diagram showing the number of homologous gene families per species. (B) Distribution of 1:1 orthologous gene in five mammals is displayed. **Figure S4.** Screening of TSGs in other four mammals. The distribution of the tissue specificity index (*τ*) and the expression levels of TSGs are showed. The dotted lines represent the value of top 20% of *τ* scores and the significance is calculated using one-sided Wilcoxon rank-sum test (*P* < 2.00 × 10^− 16^). And some genes could be expressed in more than one tissue type. (A, B) Cattle. (C, D) Sheep. (E, F) Human. (G, H) Mouse. * *P* < 0.05; ** *P* < 0.01; *** *P* < 0.001. **Figure S5.** Location distribution of porcine TSGs. Histogram showing the number of TSGs of pig on each chromosome. Scaffold represents genes whose chromosomal location is uncertain. *P* value is calculated using hypergeometric distribution test, and * *P* < 0.05; ** *P* < 0.01; *** *P* < 0.001.

**Additional file 2: Table S8.** TSGs in pig.

**Additional file 3: Table S9.** The significantly related TFs in porcine TSGs、NTSGs、LCTSGs、MCTSGs and HCTSGs.

**Additional file 4: Table S10.** Testis-specific transcripts in pig.

## Data Availability

All data generated or analyzed during this study were included in this published article and its supplementary information files. And the accession numbers corresponding to the datasets used and analysed in this study can be found in Tables S1. These data sets come from the SRA and BioProject databases of the National Center for Biotechnology Information.

## Abbreviations

A3: alternative 3′ splice sites
A5: alternative 5′ splice sites
AF: alternative first exons
AL: alternative last exons
AS: alternative splicing
dN: nonsynonymous substitution rates
dS: synonymous substitution rates
FDR: false discovery rate
FPKM: fragments per kilobase of transcript per million mapped reads
GO: Gene Ontology
GSEA: Gene Set Enrichment analysis
HCTSGs: high expression conservation TSGs
KEGG: Kyoto Encyclopedia of Genes and Genomes gene sets
LCTSGs: low expression conservation TSGs
LY: Landrace×Yorkshire pig
MC: multi-copy genes
MCL: Markov Cluster algorithm
MCTSGs: moderate expression conservation TSGs
MX: mutually exclusive exons
NTSGs: non-testis-specific genes
PCA: principal component analysis
PSI: Percent spliced in
PWMs: position weight matrices
qRT-PCR: quantitative real-time PCR
RI: retained intron
RNA-seq: RNA sequencing
SC: single-copy genes
SE: exon skipping
TFs: transcription factors
TSGs: testis-specific genes
