# Supplementary material for: Identification and characterization of male reproduction-related genes in pig (Sus scrofa) using transcriptome analysis
Source: BMC Genomics. 2020 Jun 1;21:381. doi: 10.1186/s12864-020-06790-w (PMC7268776; doi:10.1186/s12864-020-06790-w)
Supplement: Supplementary file 1 — Additional file 1: Table S1. SRA samples and project numbers of twelve organs in five mammals. Table S2. Mapping statistics of different organs of pig (Sus scrofa). Table S3. Mapping statistics of different organs of cattle (Bos taurus). Table S4. Mapping statistics of different organs of sheep (Ovis aries). Table S5. Mapping statistics of different organs of human (Homo sapiens). Table S6. Mapping statistics of different organs of mouse (Mus musculus). Table S7. Examples of identified TSGs presented in Table S8. Figure S1. Mapped ratio of mapped to the reference genome in five mammals. Figure S2. Distribution difference of protein-coding genes between testis and other organs in pig. (A-I) Quantile-Quantile (q-q) Plots represent the distribution difference of all expressed protein-coding genes (log2-transformed FPKM) between the testis and other nine organs in pig. The blue lines (y = x) mean the same distribution. Figure S3. Identification of homologous families and homologous genes in five mammals. (A) Venn diagram showing the number of homologous gene families per species. (B) Distribution of 1:1 orthologous gene in five mammals is displayed. Figure S4. Screening of TSGs in other four mammals. The distribution of the tissue specificity index (τ) and the expression levels of TSGs are showed. The dotted lines represent the value of top 20% of τ scores and the significance is calculated using one-sided Wilcoxon rank-sum test (P < 2.00 × 10− 16). And some genes could be expressed in more than one tissue type. (A, B) Cattle. (C, D) Sheep. (E, F) Human. (G, H) Mouse. * P < 0.05; ** P < 0.01; *** P < 0.001. Figure S5. Location distribution of porcine TSGs. Histogram showing the number of TSGs of pig on each chromosome. Scaffold represents genes whose chromosomal location is uncertain. P value is calculated using hypergeometric distribution test, and * P < 0.05; ** P < 0.01; *** P < 0.001. [file 12864_2020_6790_MOESM1_ESM.docx]

**Identification and characterization of male reproduction-related genes in pig (*Sus scrofa*) using transcriptome analysis**

Wenjing Yang^1^, Feiyang Zhao^2^, Mingyue Chen^1^, Ye Li^2^, Xianyong Lan^1^, Ruolin Yang^2^*, Chuanying Pan^1^*

^1^ College of Animal Science and Technology, Northwest A&F University, key Laboratory of Animal Genetics, Breeding and Reproduction of Shaanxi Province, Yangling, Shaanxi 712100, PR China;

^2^ College of Life Sciences, Northwest A&F University, Yangling, Shaanxi 712100, PR China.

* Corresponding authors: Chuanying Pan and Ruolin Yang

# Table S1. SRA samples and project numbers of twelve organs in five mammals.

| **Tissues** | ***Sus scrofa*** | ***Bos taurus*** | ***Ovis aries*** | ***Homo sapiens*** | ***Mus musculus*** |
| --- | --- | --- | --- | --- | --- |
| Testis | SRR7041980  PRJNA451072 | SRR594481  PRJNA177791 | ERR489222  PRJEB6169 | SRR4421668  PRJNA30709 | SRR5047953  PRJNA66167 |
|  | SRR7041985  PRJNA451072 | — | ERR489223  PRJEB6169 | SRR4422588  PRJNA30709 | SRR5047956  PRJNA66167 |
| Brain | SRR5470021  PRJNA384017 | SRR594473  PRJNA177791 | ERR489218  PRJEB6169 | SRR5171092  PRJNA30709 | SRR567498 |
|  | SRR5470022  PRJNA384017 | — | ERR489219  PRJEB6169 | — | SRR567499 |
| Cerebellum | ERR1824459  PRJEB19386 | — | ERR489216  PRJEB6169 | SRR3192427  PRJNA30709 | SRR5048025  PRJNA66167 |
|  | ERR1824460  PRJEB19386 | — | ERR489217  PRJEB6169 | SRR3192428  PRJNA30709 | SRR5048026  PRJNA66167 |
| Hypothalamus | SRR1022876  PRJNA225941 | SRR1178419  PRJNA239571 | ERR489198  PRJEB6169 | — | — |
|  | SRR1022884  PRJNA225941 | SRR1178420  PRJNA239571 | ERR489199  PRJEB6169 | — | — |
|  | — | SRR1178421  PRJNA239571 | — | — | — |
| Pituitary | ERR1824417  PRJEB19386 | SRR1178426  PRJNA239571 | ERR489200  PRJEB6169 | — | — |
|  | ERR1824418  PRJEB19386 | SRR1178428  PRJNA239571 | ERR489201 PRJEB6169 | — | — |
|  | — | SRR1178429  PRJNA239571 | — | — | — |
| Heart | SRR3160008  PRJNA311523 | SRR594475  PRJNA177791 | ERR489258  PRJEB6169 | SRR3192433  PRJNA30709 | SRR5047921  PRJNA66167 |
|  | SRR3160018  PRJNA311523 | — | ERR489259  PRJEB6169 | SRR3192434  PRJNA30709 | SRR5047923  PRJNA66167 |
| Liver | SRR3160009  PRJNA311523 | SRR594477  PRJNA177791 | ERR489264  PRJEB6169 | SRR3192439  PRJNA30709 | SRR5047933  PRJNA66167 |
|  | SRR3160019  PRJNA311523 | — | ERR489265  PRJEB6169 | SRR3192440  PRJNA30709 | SRR5047936  PRJNA66167 |
| Kidney | SRR3160012  PRJNA311523 | SRR594476  PRJNA177791 | — | SRR5171070  PRJNA30709 | SRR5047927  PRJNA66167 |
|  | SRR3160022  PRJNA311523 | — | — | — | SRR5047930  PRJNA66167 |
| Fat | SRR3160015  PRJNA311523 | SRR1178465  PRJNA239571 | — | SRR4422144  PRJNA30709 | SRR5048012  PRJNA66167 |
|  | SRR3160025  PRJNA311523 | SRR1178466  PRJNA239571 | — | SRR4422184  PRJNA30709 | SRR5048013  PRJNA66167 |
|  | — | SRR1178467  PRJNA239571 | — | — | — |
| Renal cortex | — | — | ERR489260  PRJEB6169 | — | — |
|  | — | — | ERR489261  PRJEB6169 | — | — |
| Skeletal muscle | SRR3160013  PRJNA311523 | SRR594479  PRJNA177791 | ERR489246  PRJEB6169 | SRR3192453  PRJNA30709 | SRR3192320  PRJNA66167 |
|  | SRR3160023  PRJNA311523 | — | ERR489247  PRJEB6169 | SRR3192454  PRJNA30709 | SRR3192322  PRJNA66167 |
| Skin | — | — | ERR489248  PRJEB6169 | SRR3192447  PRJNA30709 | SRR2927740  PRJNA301404 |
|  | — | — | ERR489249  PRJEB6169 | SRR3192448  PRJNA30709 | — |

Note: For previous research, researchers have used the data for differential expression gene analysis, cluster analysis, RNA editing and so on. Brain of mouse did not have the project number.

# Table S2. Mapping statistics of different organs of pig (*Sus scrofa*).

| **Tissues** | **Raw Reads** | **Total Reads** | **Mapped Reads** | **Mapped Ratio** |
| --- | --- | --- | --- | --- |
| Testis | 19110150 | 19110150 | 17539299 | 91.78% |
|  | 23330476 | 23330476 | 21772245 | 93.32% |
| Brain | 22206828 | 22206828 | 16628128 | 74.88% |
|  | 23721567 | 23721567 | 20676030 | 87.16% |
| Cerebellum | 14270709 | 10404702 | 9214416 | 88.56% |
|  | 18290558 | 13331152 | 11867862 | 89.02% |
| Hypothalamus | 22051471 | 22051471 | 19898128 | 90.23% |
|  | 19855236 | 19855236 | 18223703 | 91.78% |
| Pituitary | 18983256 | 13619920 | 12081991 | 88.71% |
|  | 19129791 | 13692615 | 12140952 | 88.67% |
| Heart | 27119440 | 27119440 | 24650758 | 90.90% |
|  | 21642868 | 21642868 | 19214574 | 88.78% |
| Liver | 30074231 | 30074231 | 27384036 | 91.05% |
|  | 32162589 | 32162589 | 29672805 | 92.26% |
| Kidney | 18409341 | 18409341 | 16680719 | 90.61% |
|  | 24180917 | 24180917 | 21790794 | 90.12% |
| Fat | 33462368 | 33462368 | 30369163 | 90.76% |
|  | 28663354 | 28663354 | 25832957 | 90.13% |
| Skeletal muscle | 36293178 | 36293178 | 32804641 | 90.39% |
|  | 23827661 | 23827661 | 21247884 | 89.17% |

# Table S3. Mapping statistics of different organs of cattle (*Bos taurus*).

| **Tissues** | **Raw Reads** | **Total Reads** | **Mapped Reads** | **Mapped Ratio** |
| --- | --- | --- | --- | --- |
| Testis | 107483976 | 92572729 | 86404704 | 93.34% |
| Brain | 104353205 | 91986227 | 84381621 | 91.73% |
| Hypothalamus | 28776094 | 28776094 | 26383178 | 91.68% |
|  | 31364350 | 31364350 | 29175069 | 93.02% |
|  | 30031193 | 30031193 | 27585566 | 91.86% |
| Pituitary | 27765379 | 27765379 | 25558596 | 92.05% |
|  | 31663654 | 31663654 | 29213715 | 92.26% |
|  | 31709885 | 31709885 | 28854233 | 90.99% |
| Heart | 117554231 | 94815212 | 88243221 | 93.07% |
| Liver | 103019718 | 103019718 | 90256656 | 87.61% |
| Kidney | 115720336 | 115720336 | 101551798 | 87.76% |
| Fat | 24690514 | 24690514 | 22963547 | 93.01% |
|  | 28858781 | 28858781 | 26753506 | 92.70% |
|  | 26487892 | 26487892 | 24602552 | 92.88% |
| Skeletal muscle | 113227169 | 91585728 | 83168969 | 90.81% |

# Table S4. Mapping statistics of different organs of sheep (*Ovis aries*).

| **Tissues** | **Raw Reads** | **Total Reads** | **Mapped Reads** | **Mapped Ratio** |
| --- | --- | --- | --- | --- |
| Testis | 27586347 | 21251352 | 17636485 | 82.99% |
|  | 28093276 | 21629126 | 18002757 | 83.23% |
| Brain | 22279392 | 14414543 | 12667270 | 87.88% |
|  | 22399418 | 14548714 | 12789083 | 87.91% |
| Cerebellum | 20737613 | 14242365 | 12672714 | 88.98% |
|  | 20864606 | 14356195 | 12778070 | 89.01% |
| Hypothalamus | 20033166 | 15110480 | 12885084 | 85.27% |
|  | 20711445 | 15651896 | 13337382 | 85.21% |
| Pituitary | 24368745 | 18224967 | 16374966 | 89.85% |
|  | 24364806 | 18248188 | 16406556 | 89.91% |
| Heart | 34463780 | 24087181 | 20650547 | 85.73% |
|  | 34392062 | 24060004 | 20562824 | 85.46% |
| Liver | 18609670 | 13932975 | 11188325 | 80.30% |
|  | 18880692 | 14168805 | 11375685 | 80.29% |
| Renal cortex | 22234531 | 16511151 | 13787230 | 83.50% |
|  | 22198964 | 16493355 | 13736321 | 83.28% |
| Skeletal muscle | 18319627 | 13119259 | 11101721 | 84.62% |
|  | 18283429 | 13104728 | 11061705 | 84.41% |
| Skin | 23275095 | 16495178 | 13499274 | 81.84% |
|  | 23235726 | 16472967 | 13454488 | 81.68% |

# Table S5. Mapping statistics of different organs of human (*Homo sapiens*).

| **Tissues** | **Raw Reads** | **Total Reads** | **Mapped Reads** | **Mapped Ratio** |
| --- | --- | --- | --- | --- |
| Testis | 57055746 | 57055746 | 50748695 | 88.95% |
|  | 65954194 | 65954194 | 58907318 | 89.32% |
| Brain | 28523713 | 28523713 | 23008933 | 80.67% |
| Cerebellum | 121997943 | 121997943 | 107788191 | 88.35% |
|  | 87218921 | 87218921 | 75462307 | 86.52% |
| Heart | 77191913 | 77191913 | 61975116 | 80.29% |
|  | 96496582 | 96496582 | 77657227 | 80.48% |
| Liver | 86121757 | 86121757 | 77139545 | 89.57% |
|  | 103534518 | 103534518 | 90514143 | 87.42% |
| Kidney | 28492976 | 28492976 | 20222927 | 70.98% |
| Fat | 52002764 | 52002764 | 46587761 | 89.59% |
|  | 45818006 | 45818006 | 40896591 | 89.26% |
| Skeletal muscle | 93154341 | 93154341 | 85154350 | 91.41% |
|  | 101285674 | 101285674 | 90150935 | 89.01% |
| Skin | 113729676 | 113729676 | 91289371 | 80.27% |
|  | 79756716 | 79756716 | 71485556 | 89.63% |

# Table S6. Mapping statistics of different organs of mouse (*Mus musculus*).

| **Tissues** | **Raw Reads** | **Total Reads** | **Mapped Reads** | **Mapped Ratio** |
| --- | --- | --- | --- | --- |
| Testis | 37696007 | 37696007 | 32415931 | 85.99% |
|  | 36086152 | 36086152 | 30918299 | 85.68% |
| Brain | 170695079 | 170695079 | 14148796 | 82.89% |
|  | 173161230 | 173161230 | 142404686 | 82.24% |
| Cerebellum | 150974801 | 150974801 | 120884054 | 80.07% |
|  | 144705041 | 144705041 | 119436755 | 82.54% |
| Heart | 39038956 | 39038956 | 32568493 | 83.43% |
|  | 41443334 | 41443334 | 35454251 | 85.55% |
| Liver | 30633616 | 30633616 | 26162924 | 85.41% |
|  | 31913472 | 31913472 | 26559525 | 83.22% |
| Kidney | 40542680 | 40542680 | 33579277 | 82.82% |
|  | 38866777 | 38866777 | 32838098 | 84.49% |
| Fat | 40166941 | 40166941 | 34908409 | 86.91% |
|  | 40067281 | 40067281 | 33249920 | 82.99% |
| Skeletal muscle | 26015261 | 26015261 | 22341653 | 85.88% |
|  | 27951433 | 27951433 | 24049165 | 86.04% |
| Skin | 55801575 | 55801575 | 49390360 | 88.51% |

# Table S7. Examples of identified TSGs presented in Table S8.

| **Genes** | **Identifier** | **Source (PMID)** |
| --- | --- | --- |
| *HSD17B3* | Pig | Chen et al. (31550505) |
| *CYP11A1* | Pig | Robic et al. (27436769) |
| *Stra8* | Pig | Wang et al. (25029539) |
| *PGK2* | Pig | Chen et al. (15599558) |
| *CIB4* | Sheep | Mohammadabadi et al. (28902924) |
| *DMRT1* | Sheep | Li et al. (29174386) |
| *TEX14* | Mouse | Greenbaum et al. (16549803) |
| *SUN5* | Mouse | Shang et al. (28945193) |
| *KLHL10* | Mouse | Yan et al. (15136734) |
| *PRSS37* | Mouse | Shen et al. (23553430) |
| *PRSS55* | Mouse | Shang et al. (30032357) |
| *PRSS54* | Mouse、Human | Holcomb et al. (31403672) |
| *TEX35*(*Tsc24*) | Mouse、Human | Tang et al. (17077512) |
| *ACRV1* | Mouse、Human | Tang et al. (21488928) |
| *SEPT12* | Mouse、Human | Lin et al. (19359518) |
| *CFAP65* | Human | Zhang et al. (31571197) |
| *ZMYND15* | Human | Ayhan et al. (24431330) |
| *DAZL* | Human | Hashemi et al. (30054974) |
| *TNP1* | Human | Hashemi et al. (30054974) |
| *TEPP* | Human | Bera et al. (14652002) |

**
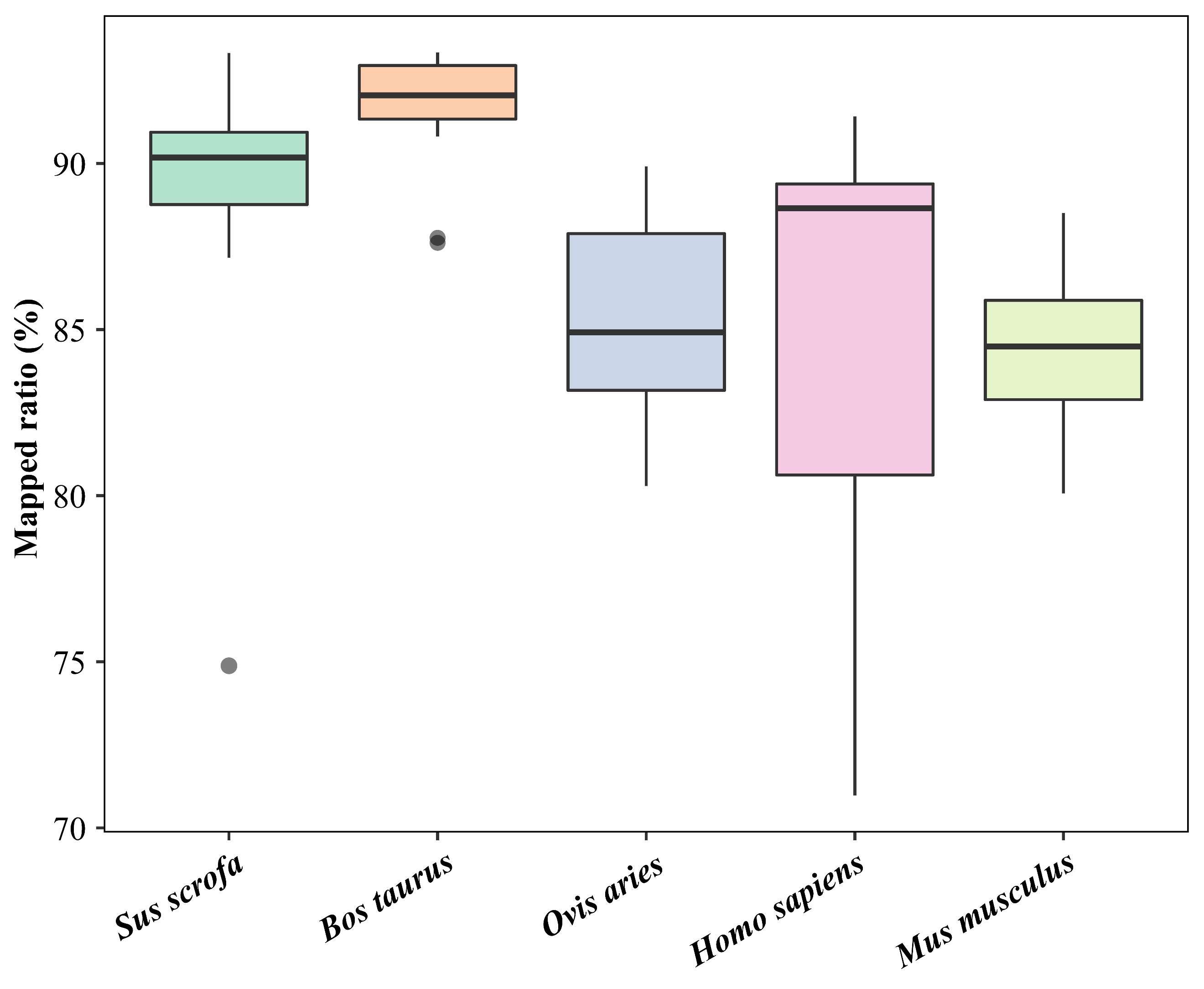
**

**Figure S1.** Mapped ratio of mapped to the reference genome in five mammals.

**
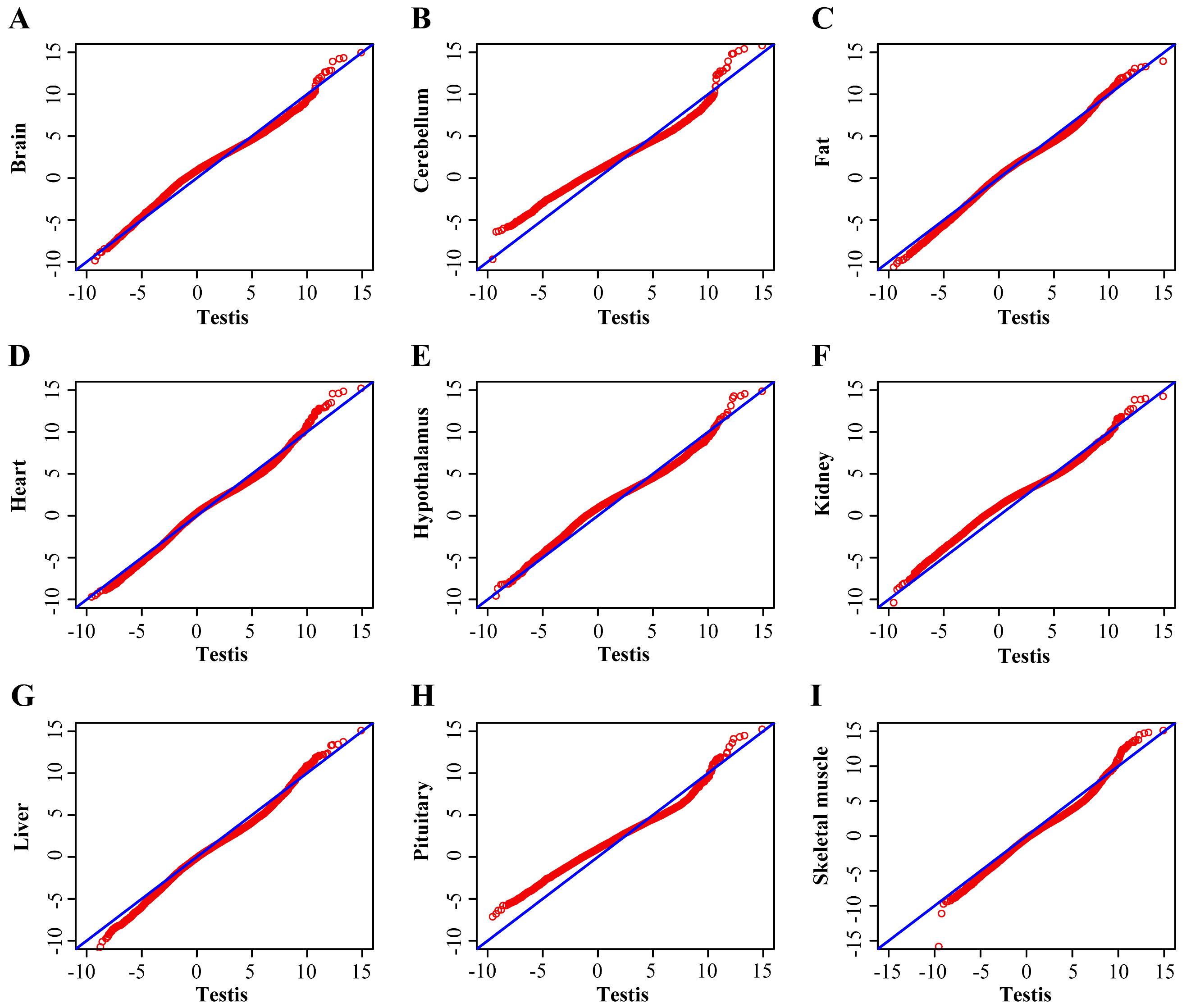
**

# Figure S2. Distribution difference of protein-coding genes between testis and other organs in pig. (A-I) Quantile-Quantile (q-q) Plots represent the distribution difference of all expressed protein-coding genes (log2-transformed FPKM) between the testis and other nine organs in pig. The blue lines (y=x) mean the same distribution.

**
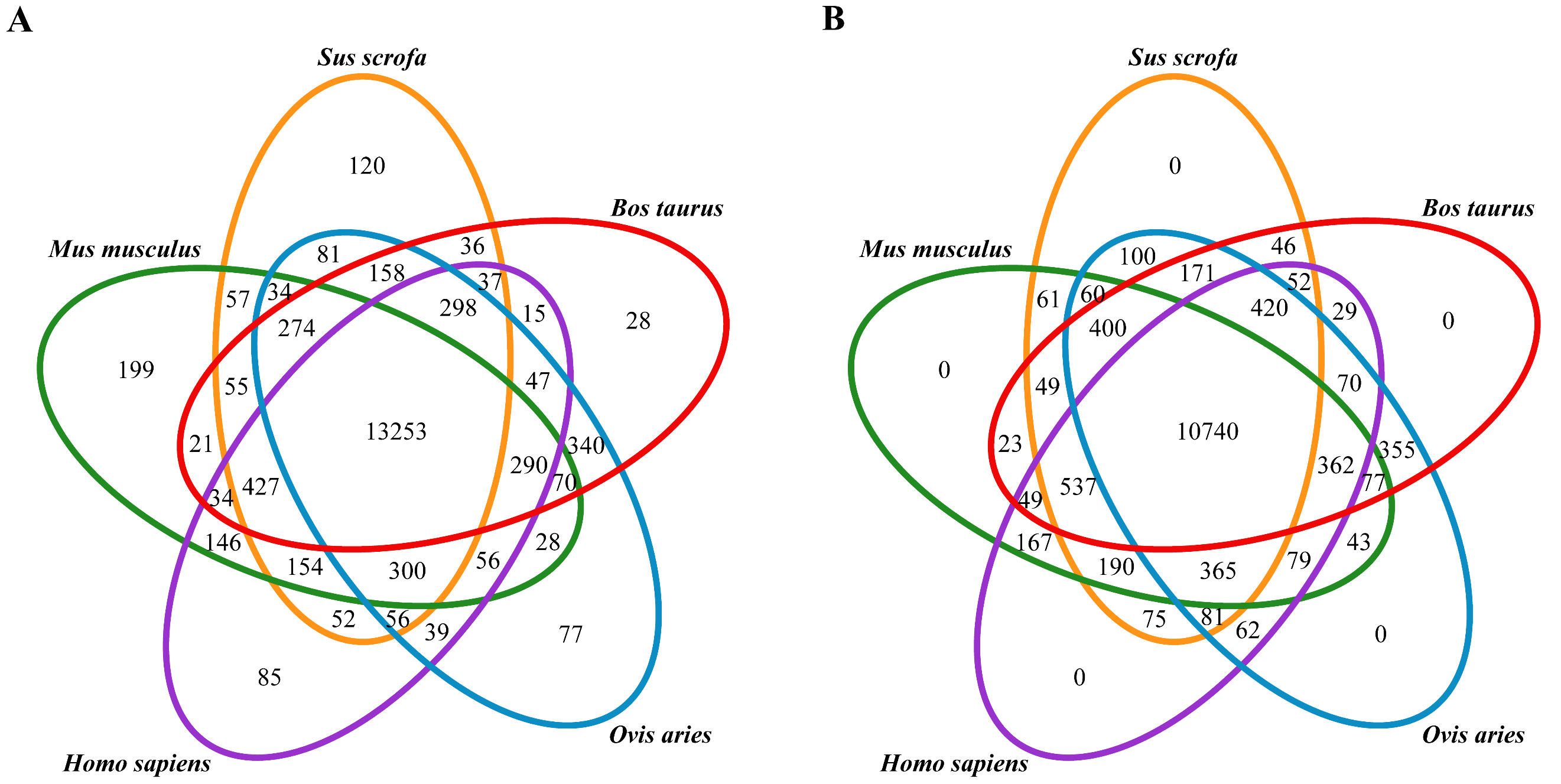
**

# Figure S3. Identification of homologous families and homologous genes in five mammals. (A) Venn diagram showing the number of homologous gene families per species. (B) Distribution of 1:1 orthologous gene in five mammals is displayed.

**
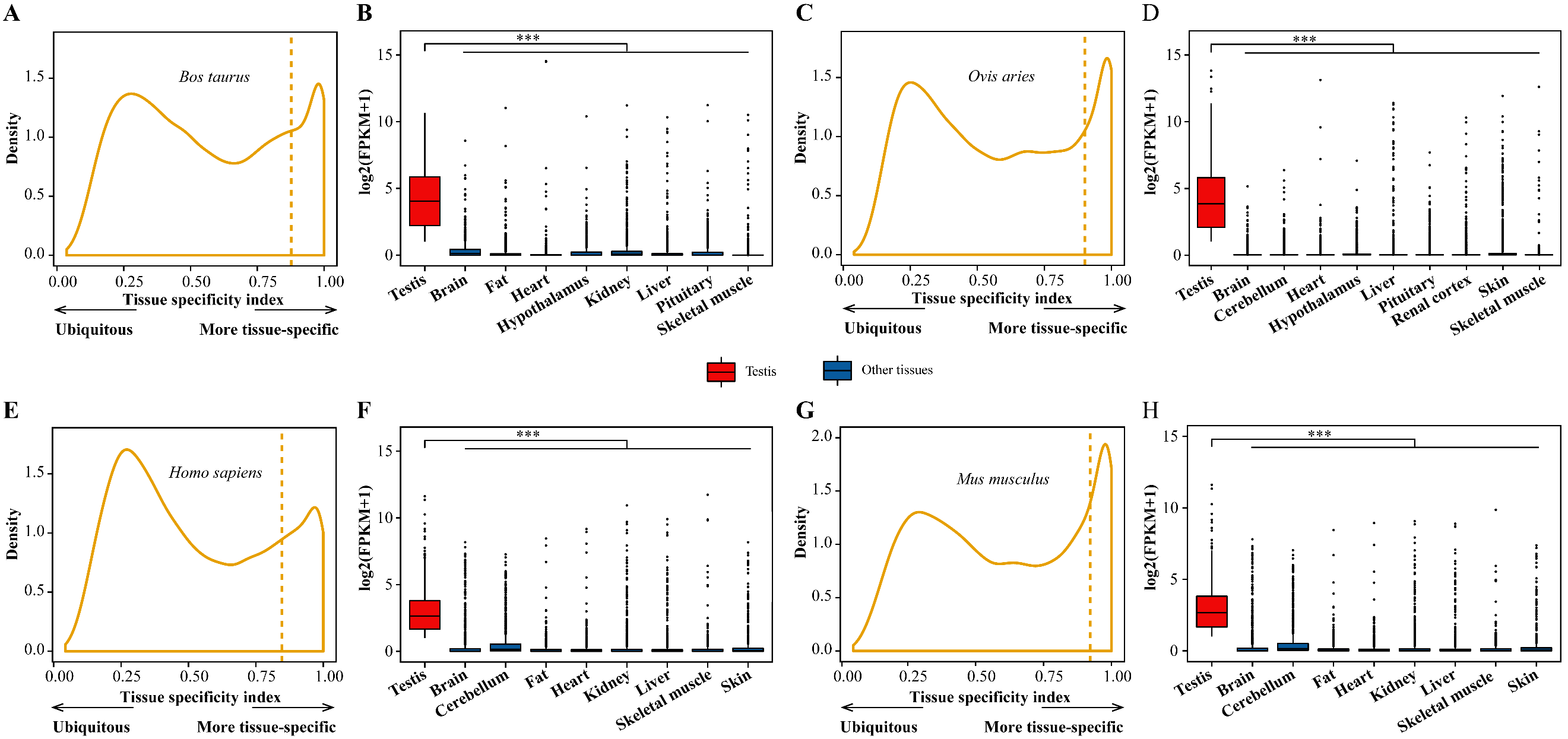
**

# Figure S4. Screening of TSGs in other four mammals. The distribution of the tissue specificity index (*τ*) and the expression levels of TSGs are showed. The dotted lines represent the value of top 20% of *τ* scores and the significance is calculated using one-sided Wilcoxon rank-sum test (*P* < 2×10^-16^). And some genes could be expressed in more than one tissue type. (A, B) Cattle. (C, D) Sheep. (E, F) Human. (G, H) Mouse. * *P* < 0.05; ** *P* < 0.01; *** *P* < 0.001.


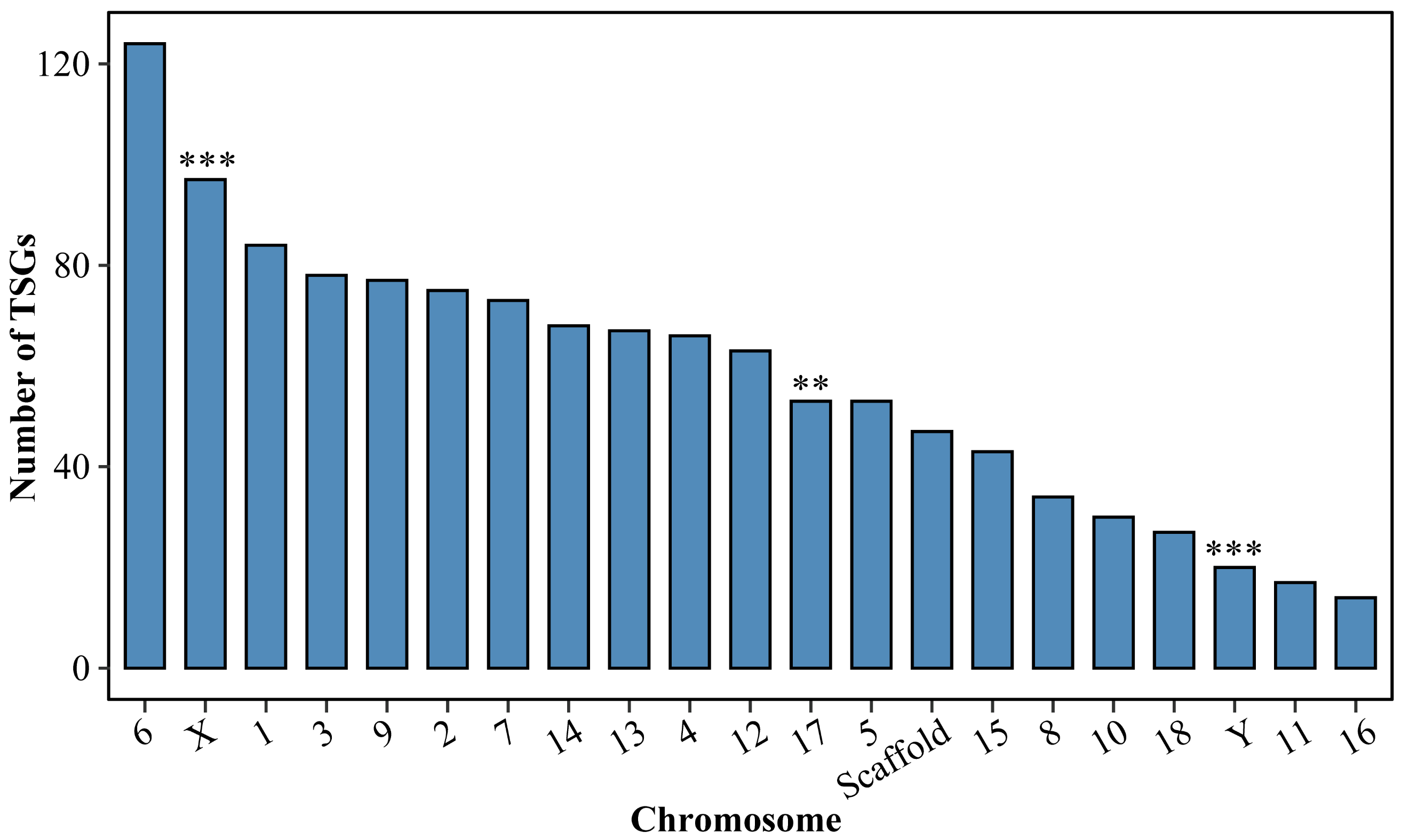


# Figure S5. Location distribution of porcine TSGs. Histogram showing the number of TSGs of pig on each chromosome. Scaffold represents genes whose chromosomal location is uncertain. *P* value is calculated using hypergeometric distribution test, and * *P* < 0.05; ** *P* < 0.01; *** *P* < 0.001.
